# Imaging Report With Radiologic Criteria for Differentiation: Avulsion Fracture or Os Triquetrum Secundarium?

**DOI:** 10.7759/cureus.93942

**Published:** 2025-10-06

**Authors:** Lyubomir Gaydarski, Kristina Petrova, Lukasz Olewnik, Ingrid C Landfald, Maria Piagkou, Mugurel C Rusu, Boycho Landzhov, Georgi P Georgiev

**Affiliations:** 1 Department of Anatomy, Histology, and Embryology, Medical University-Sofia, Sofia, BGR; 2 VARIANTIS Research Laboratory, Department of Clinical Anatomy, Mazovian Academy, Płock, POL; 3 Department of Clinical Anatomy, Mazovian Academy, Płock, POL; 4 Department of Anatomy/Oral Surgery, National and Kapodistrian University of Athens, Athens, GRC; 5 Department of Anatomy, Faculty of Dentistry, “Carol Davila” University of Medicine and Pharmacy, Bucharest, ROU; 6 Department of Orthopaedics and Traumatology, University Hospital Queen Giovanna-ISUL, Medical University of Sofia, Sofia, BGR

**Keywords:** conservative treatment, differential diagnosis, os intermedium antebrachii, os triangulare carpi, wrist ossicles

## Abstract

Accessory ossicles of the wrist are uncommon bone variants that can be mistaken for avulsion fractures, potentially leading to unnecessary tests, procedures, and radiation exposure. We report a case of a 19-year-old male who presented after a motor vehicle collision with bilateral wrist pain and swelling. Radiographs of the right wrist were normal. Imaging of the left wrist demonstrated a small, oval ossicle located between the triquetrum and the ulnar styloid process. The ossicle displayed smooth cortical margins, a preserved cortical-medullary ratio, and a distinct radiolucent gap from adjacent carpal bones, features consistent with an os triquetrum secundarium (OTS). The patient denied prior wrist trauma, and no changes in the normal range of motion in the wrists were established. The diagnosis was reached by correlating the clinical history with radiographic findings. Conservative management, including immobilization, nonsteroidal anti-inflammatory drugs, cryotherapy, and physiotherapy, resulted in complete symptom resolution within two weeks. This case underscores the importance of including OTS in the differential diagnosis of ulnar-sided wrist pain and suspected avulsion fractures, applying established imaging criteria to avoid misdiagnosis, and favoring nonoperative treatment when there is no associated triangular fibrocartilage complex injury.

## Introduction

Accessory ossicles (AOs) of the upper limb, particularly within the carpal region, are rare osseous variants with important clinical implications. Their significance lies not in inherent pathology but in the potential for misdiagnosis, especially in trauma settings where they can be mistaken for acute fractures [1-3]. To date, over 25 distinct accessory wrist bones or accessory carpal ossicles (ACOs) have been described in the anatomical and radiological literature, some documented as early as the early 20th century [4,5].

One such ossicle, the os triquetrum secundarium (OTS), also known as the os triangulare carpi or os intermedium antebrachii, is located between the triquetrum and the ulnar styloid process [2,3]. Its prevalence ranges from 0.1% to 2.4%, varying by population cohort and imaging modality employed [2,6]. This variant was also listed in Bergman’s Comprehensive Encyclopedia of Human Anatomic Variation, but without any reported incidence rate or further morphological details about the ossicle. It was, however, noted that such a variant is commonly mistaken for an ulnar styloid fracture [7].

Although typically asymptomatic and identified incidentally on imaging studies, AOs can become clinically significant in the context of acute trauma or repetitive mechanical stress. In such cases, they may mimic avulsion fractures and lead to unnecessary diagnostic workup, including advanced imaging or even invasive intervention, ultimately contributing to increased healthcare burden and patient radiation exposure [2].

This imaging report highlights a rare case of unilateral OTS, incidentally found during radiographic evaluation after a motor vehicle collision. The case emphasizes the importance of including OTS in the differential diagnosis of wrist pain and trauma, as well as the usefulness of established radiographic criteria to differentiate developmental ossicles from fracture fragments. Early recognition can facilitate conservative management and help avoid unnecessary treatment.

## Case presentation

A 19-year-old male presented to the emergency department following a motor vehicle collision, reporting acute bilateral wrist pain and swelling. Physical examination revealed mild edema over the carpal regions bilaterally, without any significant motor deficits. There was no tenderness or swelling localized over the ulnar styloid or distal ulna. Range of motion was normal in both wrists. Grip strength was preserved. The triangular fibrocartilage complex (TFCC) compression test was negative. Neurovascular assessment demonstrated intact distal perfusion and sensation in both upper extremities. The patient was consulted with general surgery, neurosurgery, orthopedic surgery, and anesthesiology in accordance with our institutional protocol for managing emergency patients involved in motor vehicle collisions.

**Figure 1:**
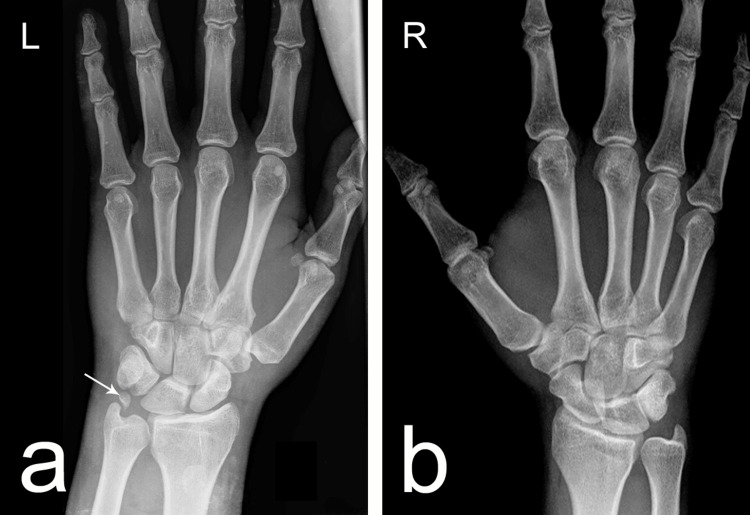
Anteroposterior radiographs of the wrists. (a) The left wrist demonstrates the presence of an os triquetrum secundarium (OTS), indicated by the white arrow. The ossicle appears as a discrete, ovoid structure located between the triquetrum and the ulnar styloid process, with smooth cortical margins, a preserved cortical-to-medullary ratio, and a distinct radiolucent gap from adjacent bones—features consistent with a developmental accessory ossicle. (b) The right wrist shows typical carpal anatomy without accessory ossicles or osseous abnormalities.

Standard radiographs were obtained for both wrists (Figure 1). The right wrist (Figure 1B) showed no osseous abnormalities or accessory ossicles. In contrast, the left wrist (Figure 1A) demonstrated a discrete, ovoid, bean-shaped ossicle located between the triquetrum and the ulnar styloid process. The ossicle measured approximately 0.5 × 0.3 cm, with smooth, well-defined cortical margins, a preserved cortical-to-medullary ratio, and a distinct radiolucent gap separating it from adjacent carpal bones. These imaging characteristics were consistent with a developmental accessory ossicle rather than an acute avulsion fragment.

The patient denied any prior wrist or upper extremity injuries during childhood. After correlating the clinical, radiographic, and historical data, no further imaging (CT or MRI) was performed, and a diagnosis of unilateral OTS was established. Conservative management was initiated, including wrist immobilization, a 10-day course of nonsteroidal anti-inflammatory drugs (NSAIDs), localized cryotherapy, and a structured physiotherapy program. At the two-week follow-up, the patient reported complete resolution of symptoms and had returned to full wrist function without residual pain or swelling.

## Discussion

The present imaging report of a unilateral OTS, found incidentally on wrist radiographs after a motor vehicle accident, adds to the limited literature on this rare ACO. While usually benign, OTS can pose diagnostic challenges in trauma settings, where it may closely resemble acute fracture fragments. Our review of published epidemiological data identified six studies that reported on OTS prevalence, summarized in Table 1. Two early studies from the 20th century, Bogart [6] and O’Rahilly [4], provided foundational descriptions of ACOs, although specific data for OTS were often incomplete. More recent research, including Amar et al. [8], Gursoy et al. [2], Atay et al. [9], and Kose et al. [3], used both conventional radiography and computed tomography (CT) scans to evaluate large groups. In these studies, reported OTS prevalence ranged from 0.1% to 2.4% based on radiographs and 1.3% when assessed via CT scans. The higher detection rate in CT-based studies, like Kose et al. [3], highlights how imaging modality influences prevalence estimates, suggesting some cases may go unnoticed with plain radiography. Table 1 shows that the overall prevalence of ACOs varies widely, from 0.4% in early 20th-century surveys to nearly 10% in some modern series, likely due to improvements in imaging quality, population differences, and varying diagnostic criteria. This variability supports the idea that OTS may be underreported or misclassified, especially in the acute trauma setting (Table 1).

**Table 1:** Summary of epidemiological studies reporting the prevalence of accessory carpal ossicles (ACOs) with specific reference to the os triquetrum secundarium (os triangulare).

Study (author, year)	Patient cohort size (n)	Imaging modality	Overall ACOs prevalence (%)	Os triangulare prevalence (%)
Bogart (1932) [6]	1,452	Radiograph	0.4	0.1
O’Rahilly (1953) [4]	743	Radiograph	1.6	Not specified
Amar et al. (2011) [8]	442	Radiograph	2.5	0.7
Gursoy et al. (2021) [2]	1,146	Radiograph	9.7	2.4
Atay et al. (2023) [9]	500	Radiograph	1.6	1.6
Kose et al. (2024) [3]	2,213	CT	7.1	1.3

The cause of OTS is still debated. Proposed mechanisms include failure of congenital ossification of a secondary ossification center, developmental separation of the epiphyseal plate, and post-traumatic ossification after an unrecognized avulsion injury. Among these, the congenital theory remains the most widely accepted [2,10], viewing OTS as a benign anatomical variation rather than a result of injury.

In trauma scenarios, distinguishing accessory ossicles from acute fracture fragments is crucial to prevent unnecessary interventions. Radiological criteria from Kunc et al. highlight a regular ovoid shape, smooth cortical margins, and maintained cortical-medullary continuity [11]. Gaydarski et al. additionally suggest that the absence of early-life trauma, the presence of a radiolucent gap between the ossicle and nearby bones, and the lack of fibrotic bridging are essential to differentiate OTS from fracture [12]. Our case met all these criteria, confirming the diagnosis. Although most OTS cases remain asymptomatic, they can contribute to pathology through two main mechanisms. First, as a space-occupying structure, an ossicle can change ulnocarpal joint biomechanics, increasing the risk of degenerative changes in the TFCC, often within the spectrum of ulnocarpal impaction syndrome [13,14]. Second, an existing TFCC injury may destabilize the joint, leading to secondary impingement and symptom development in a previously silent OTS [15,16]. Given the complexity of ulnar-sided wrist pain, often called the wrist’s “black box” because of its overlapping anatomical pain sources, diagnosis requires a systematic approach [17,18]. Table 2 outlines the different possible diagnoses for ulnar-sided wrist pain, highlighting the clinical history, key physical exam maneuvers, and imaging findings that help distinguish symptomatic OTS from other causes such as TFCC tears, ulnocarpal impaction, extensor carpi ulnaris (ECU) tendinopathy, lunotriquetral ligament injury, pisotriquetral arthritis, hook of hamate fracture, and ulnar nerve compression. This organized comparison shows that although several conditions have similar symptoms, careful correlation of clinical and imaging data can narrow down the diagnosis and avoid misinterpretation (Table 2).

**Table 2:** Differential diagnosis of ulnar-sided wrist pain and accessory ossicle presentation based on clinical history, physical examination, and imaging findings. OTS: os triquetrum secundarium; TFCC: triangular fibrocartilage complex; DRUJ: distal radioulnar joint; ECU: extensor carpi ulnaris; VISI: volar intercalated segment instability; MRI: magnetic resonance imaging; MR arthrogram: magnetic resonance arthrography; CT: computed tomography.

Condition	Key history/symptoms	Provocative physical exam maneuvers	Key imaging findings
Symptomatic variant bone (e.g., OTS)	Chronic, activity-related ulnar pain; may include clicking. Often, no trauma history or repetitive twisting [12].	Point tenderness in the ulnocarpal space (fovea); pain with ulnar deviation and axial load (ulnocarpal stress test) [18].	Radiographs: Well-defined, smooth margins; preserved cortical–medullary architecture; radiolucent separation from carpal bones. MRI: Hyaline cartilage lining; absence of bridging fibrous tissue [11,12].
TFCC tear	Ulnar pain with clicking or popping, grip weakness, and wrist instability, often preceded by a fall or torsional trauma [19].	Fovea tenderness; positive TFCC compression test or piano key test for DRUJ instability [19].	X-ray: May be normal or show positive ulnar variance. MRI: Gold standard for TFCC perforation or abnormal signal [19].
Ulnocarpal impaction syndrome	Chronic ulnar wrist pain worsened by pronation and grip-loading [19].	Diffuse ulnar tenderness; pain with ulnocarpal stress test [19].	X-ray: Positive or neutral ulnar variance. MRI: TFCC thinning or tear, chondromalacia of lunate/triquetrum, subchondral cysts [19].
ECU tendinopathy/subluxation	Dorsal-ulnar wrist pain along the extensor carpi ulnaris tendon path; snapping or clicking with rotation [17,19].	Tenderness over the 6th dorsal compartment; pain with resisted ulnar deviation; ECU synergy test [17,19].	X-ray: Usually normal. MRI/ultrasound: ECU tendon thickening, sheath fluid, or subluxation on dynamic imaging [17,19].
Lunotriquetral (LT) ligament injury	Ulnar-sided wrist pain with weakness or “clunking” sensation [19].	Tenderness over the LT interval; positive ballottement (Reagan’s) test and shear test [17,19].	X-ray: May show VISI deformity. MRI/MR arthrogram: LT ligament tear or attenuation [17,19].
Pisotriquetral (PT) arthritis	Localized pain over the pisiform, worsened by wrist flexion and ulnar deviation [17,19].	Tenderness over the pisiform; pain with grinding maneuvers [17,19].	X-ray (oblique view): Joint space narrowing, sclerosis, or osteophytes of the PT joint [17,19].
Hook of hamate fracture	Hypothenar pain, often in athletes (e.g., golfers, racquet sports), may include ulnar nerve symptoms [17,19].	Tenderness over the hook of the hamate; positive Hook of Hamate Pull Test [17,19].	X-ray: May require carpal tunnel view. CT: Preferred for occult fracture detection [17,19].
Ulnar nerve compression	Tingling or numbness in the ring and small fingers; possible intrinsic hand weakness [13,16].	Positive Tinel’s sign at Guyon’s canal or cubital tunnel; sensory deficits; intrinsic muscle [13,16].	MRI/ultrasound: May reveal compressive lesions (e.g., ganglion). Nerve conduction studies: Diagnostic [13,16].

Management of OTS depends on symptom severity and associated pathology. Conservative measures, such as immobilization, NSAIDs, cryotherapy, and physiotherapy, are suitable for asymptomatic or mildly symptomatic cases, as demonstrated here [1]. Surgical excision, either open or arthroscopic, is reserved for cases where pain persists, function is limited, or there is significant structural pathology [19]. In our patient, symptoms resolved completely within two weeks of conservative treatment, highlighting that nonoperative management is both safe and effective for incidentally detected OTS without accompanying injury.

Limitations

The main limitation of this report is that it is based on a single case, which limits the generalizability of the findings to broader populations. Additionally, the absence of advanced imaging, such as MRI, means that subtle soft-tissue injuries, especially to the TFCC, could not be completely ruled out. Our epidemiological overview also depends on studies with different methodologies, imaging techniques, and diagnostic criteria, which may lead to inconsistencies in reported prevalence rates. Future multicenter, imaging-based research could better determine the true incidence, clinical importance, and best management approaches for OTS.

## Conclusions

The present case report emphasizes the clinical importance of recognizing OTS as a rare but significant ACO. Awareness of its radiological features, such as a regular ovoid shape, smooth cortical margins, preserved medullary architecture, and separation from adjacent bones, is crucial for distinguishing it from acute fracture fragments in trauma cases. Although typically asymptomatic, OTS can occasionally cause ulnar-sided wrist pain, especially when associated with TFCC pathology or ulnocarpal impaction. A systematic diagnostic approach, combining imaging and clinical examination, can help prevent misdiagnosis and unnecessary procedures. In this patient, conservative treatment led to complete symptom resolution, reinforcing that nonoperative management remains the preferred approach for incidentally found, asymptomatic OTS.
